# The role of consultation-liaison psychiatry In the general hospital

**Published:** 2008-11-15

**Authors:** Ene Sorin

**Affiliations:** * Bagdasar-Arseni Emergency Hospital

**Keywords:** consultation-liaison psychiatry, general hospital, organic-psychiatric co-morbidity

## Abstract

This article reviews some of the recent data concerning Consultation-Liaison Psychiatry in a general hospital. As the organic medical conditions have a major impact on the one’s quality of life and self-awareness as an ill-person, very often a psychiatric disorder appears as co-morbidity. It is the responsibility of the psychiatrist to recognize and to treat these psychiatric conditions, and also to develop good work-relationships with the patients and the clinical team.

Consultation-Liaison Psychiatry (CLP) is the part of psychiatry related to the study of co-morbidities between medical general conditions (located on the third axis in the international taxonomies) and psychiatric conditions (coded on the first and the second axis). The role of CLP is that of a link between psychiatry and other medical fields. Thus, the consultation-liaison psychiatrist is the psychiatry’s ambassador in the general hospital.

Any medical condition has a significant impact on the human psychic life, first because one is psychologically aware of being ill and he/she is acting as being ill, then because the impact the general medical condition has on the life’s quality with respect to social, familial and professional activity. Furthermore, the medical and/or surgical treatment contributes to the patient’s lack of comfort, due to the medication’s side-effects or post-operatory sequelae respectively (*Hays et al. 1995, Druss et al. 1999*).

The studies on patients entering in a G.P.’s office showed at least 25% of them have a psychiatric problem (*Johnson et al. 1992*). Perhaps this group of patients is even larger, but they are not properly diagnosed, due to the next reasons:

• The general medical examination seldom includes precise questions about the psychic status of the patient.

• A few patients are prepared to have a statement regarding their own psychiatric symptoms or to accept a psychiatric diagnosis.

An estimated percent of 21-46% of the patients hospitalized in non-psychiatric settings have a psychiatric disorder. Mental disorders’ prevalence is about 25-50% with chronic medical conditions and less than 18% in those with medical conditions (*Vincze et al. 2004*).

Lifetime prevalence of mental disorders in patients with chronic medical conditions is 42% (most frequent: drugs abuse, mood disorders and anxiety disorders) and 33% in those without chronic physical disabilities. In the group of patients with short-term medical or surgical conditions, 30-60% of them presented also a psychiatric condition (*Katon, Gonzales, 1994*).

Patients hospitalized in medical departments have a significant greater psychiatric morbidity than in general population: delirium (15-30 times more), depression (2-3 times more), panic disorder and somatization (10-20 times more) drug abuse (2-3 times more). Thus, the general hospital is an excellent setting for the psychiatric screening. The only condition is to have a team for CL psychiatric consultations.

In the absence of such a team, less than 50% of the patients with psychiatric conditions are diagnosed and treated. Often depression is under-diagnosed and the treatments are under-dosed or misused (*Ni Mhaolain et al. 2008*). A Hungarian study shows that the use of antidepressants has a low frequency, as anxiolytics are commonly used (without proper indication) in depression (*Vincze et al. 2004*).

Even when depression is recognized, the rate of referrals to a psychiatrist is low (*Imam et al. 2007, Abidi et al. 2003*), in an Australian study being as low as 2.5% of all hospital admissions (*Ellen et al. 2006*).

Furthermore, psychiatric morbidity is associated with a high rate of request of medical services. Half of those who often require medical attention have also a psychiatric condition: recurrent depression or dysthymia (40%), anxiety disorders (22%), somatoform disorders (20%), panic disorder (12%), alcohol or other substances-related problems (5%) (*Fink et al. 1999*).

Depressive patients use three times more medical resources than non-depressive ones, the medical costs are twice higher in depressive patients and they require seven times more frequently the emergency services (Druss et al. 1999). Patients with panic disorder require attention in the emergency room 10 times more often than those without panic disorder, as 70% of them undergo at least 10 medical examinations until the correct diagnosis is made (*Katon et al. 1990*).

Medical costs are two times higher in patients with alcohol abuse or dependence than those without alcohol-related problems. Furthermore, 25-50% of the alcohol-related problems are not recognized in the emergency room (*Regier et al. 1993*). Similar differences are recorded in patients with anxiety disorders versus non-anxious patients.

Hospital’s costs in patients with cardiologic-psychiatric co-morbidity are four times higher than in patients with cardiac conditions only (*Schrader et al. 2005*).

A similar situation is recorded in orthopedic departments, where CLP intervention can dramatically reduce the hospitalization’s costs (*Strain et al. 1991*).

There is a high correlation between a longer hospitalization and depression or personality disorders; the same group of patients have greater re-admission rate for the next four years after the first admission (*Saravay, Lavin 1994, Aoki et al. 2003*).

On the contrary, if the depression is recognized and adequately treated while patient is in the hospital, the outcome is significantly better, the hospitalization is shorter, and the re-admission rate is lower (*Strain et al. 1991*). CLP is also important in the care of outpatients with organic co-morbidities, as there is a high rate of unrecognized depression in the general practice’s diagnosing process (*Consoli, 2003*).

This fact correlates with the G.P.’s tendencies to consider they are skilled in recognizing and treating psychiatric conditions; concomitantly, G.P.s tend to consider the CLP has the main role in advising on psycho-social issues (*Doron et al. 2003*).

Nowadays, CLP hasn’t a recognized training in EU, but its necessity in the general hospital is more and more acknowledged. In 2007, the European Association of CLP and Psychosomatics had organized a workgroup for consensus. The results in next issues were obtained:

• all residents in psychiatry should have a training in CLP work of minimum 6 months

• trainees should study:

-assessment of psychiatric disorders in somatically-ill patients;

-crisis intervention for somatically-ill patients;

-psychopharmacology in somatically-ill patients;

-communication with somatically-ill patients and with medical staff;

-organization of CLP service in the general hospital (*Sollner, Creed 2007*).

Even more, there are opinions about the necessity of a professional web in order to assure a proper care for patients with physical-psychiatric co morbidities (*Smith 2003*).

Though, there are worldwide concerns regarding the psychiatry’s role in the general hospital, as the recent trends in health care have a strong impact on the reimbursement of psychiatric assistance, particularly in certain countries (*Lipsitt 2003*).

As a conclusion, the consultation-liaison psychiatrist is a very useful part of the clinical team in approaching and treating patients with general medical conditions. Ignoring the mental level of the organic disturbance exposes the patient to the risk of untreated depression and anxiety, prolongs the suffering and affects the outcome of the organic condition.
